# #Gymlad - young boys learning processes and health-related social media

**DOI:** 10.1080/2159676X.2019.1673470

**Published:** 2019-10-07

**Authors:** Victoria Goodyear, Mikael Quennerstedt

**Affiliations:** a School of Sport, Exercise and Rehabilitation Sciences, University of Birmingham, Birmingham, United Kingdom of Great Britain and Northern Ireland; b School of Health and Medical Sciences, Örebro University, Örebro, Sweden

**Keywords:** Workout, gym, Instagram, Snapchat, feedback, praise, likes

## Abstract

Recent systematic reviews identify that the factors mediating and/or moderating the relationship between social media and health outcomes are sparse. There have also been few attempts to analyse gender specific uses of social media. This paper investigated young boys health-related learning in relation to social media. Data were generated from class activities and interviews and from a large data set that included 1346 young people. The approach to the empirical data adopted was Practical Epistemology Analysis (PEA). The findings reveal two main purposes of young boys engagement with social media: (i) communicating with friends, and (ii) accessing health-related information. Irony and humour were central learning mechanisms used by young boys to participate within health-related social media, and in a way that enabled them to engage with, uphold, and handle health discourses associated with masculinity – such as being ripped – without fear of ‘literal’ peer ridicule and within a context of acceptable ‘banter’. There was evidence that young people were critical users and generators of social media, who were clearly thinking through what they see, do, and use online. Hence, this paper provides a fresh evidence-based perspective on the potentially positive role of social media as a health-related learning resource. PEA is illustrated as a new methodological approach for investigating learning in the context of social media. The evidence generated can be used to inform future evaluations of social media use, the design of educative support for young people, and guidance and training for key stakeholders.

A considerable amount of international literature has been published on the relationship between young people, social media, and health (Dickinson et al. 2019; Orben and Przybylski 2019; Stiglic and Viner 2019). The existing body of evidence suggests that social media has a powerful influence on young people’s health-related knowledge and behaviours, in areas including physical activity, diet/nutrition, body image, sleep and mental health (Booker, Kelly, and Sacker 2018; Burnette, Kwitowski, and Mazzeo 2017; Holmberg et al. 2018; Kelly et al. 2019). So far, however, the relationship between young people’s uses of social media and their health has not been investigated in ways that provide rigorous outcomes (Dickinson et al. 2019; Orben and Przybylski 2019). In particular, recent systematic reviews identify that the factors mediating and/or moderating the relationship between social media and health outcomes are sparse (Dickinson et al. 2019; Orben and Przybylski 2019). It is also apparent that there have been few attempts to analyse sub-sets of populations, such as gender specific uses of social media (Booker, Kelly, and Sacker 2018; Dickinson et al. 2019; Kelly et al. 2019). As a result, we have a limited understanding of what aspects of young people’s uses of social media influence health-related knowledge and behaviours, and why (Dickinson et al. 2019; Stiglic and Viner 2019).

This paper contributes to knowledge about the relationship between young people, social media and health by offering in-depth insights into adolescent boys’ learning in relation to health-related social media. Learning is a complex matter with different theories providing different answers regarding what learning is and how people learn (Quennerstedt, Öhman, and Armour 2014). In this paper we use a perspective where learning is approached as a relation between the individual’s situated experiences and the institutional context where new relations contribute to how the individual makes ‘the world’ understandable in a certain situation extending possibilities for further action (Klaar and Öhman 2012).

The specific and original focus is on understanding, from the perspectives and experiences of adolescent boys, *how young people learn* in relation to social media, and how ways of learning influence the route learning takes. Using a rigorous mixed method iterative design, for the wider project (see Goodyear and Armour 2019), data were generated from a large data set that included 1346 young people. The approach to the empirical data adopted was one of Practical Epistemology Analysis (PEA). By analysing young people’s descriptions of their experiences and engagement with health-related social media we can accordingly say something about *how young people learn and what direction this learning takes*. In turn, this paper provides evidence to inform the development of new ways of investigating, evaluating and understanding the impacts of social media on young people’s health, and this evidence is relevant to researchers, policy makers, schools, and health and education professionals/practitioners. The evidence presented also provides information that will be important for teachers and parents/guardians to help them better understand how to engage with and respond to young people’s contemporary needs.

## Young people, social media and health-related learning

Most existing research on young people, social media and health has investigated the relationship between screen time or the type of social media sites that young people use (e.g. Instagram, Facebook) and health outcomes (Goodyear and Armour 2019, Burnette, Kwitowski, and Mazzeo 2017). Even though most of the associations are reported to be weak, findings suggest that screen time and social media use is associated with a variety of health harms, such as adiposity, unhealthy diet, obesity, depressive symptoms and quality of life (Orben and Przybylski 2019; Stiglic and Viner 2019). Several emerging lines of research have also focused on the offline factors that influence young people’s uses of social media, such as peers, family members and schools (Livingstone and Sefton-Green 2016; Rich 2019). While most of that research is often not directly related to health, there is evidence to suggest that peers, family members and schools are powerful influencers over the types of digital health-related content that young people engage with and use (Goodyear, Armour and Wood2019a Rich 2019). Existing research has therefore tended to investigate *what* health-related outcomes are associated with social media, and explain *why* young people are influenced to use health-related social media.

We would argue that previous studies have not dealt sufficiently with *how* young people’s engagement with social media influences health-related knowledge and behaviours. By investigating *what* and *why* young people engage with health-related social media the analytical gaze of current research has been too far ‘zoomed out’ to provide the necessary depth and detail required to explain the factors mediating or moderating health outcomes (Larsson and Quennerstedt 2016 p. 66). Notably, there is little robust data that explains the transactional processes involved in the acquisition of skills, knowledge and behaviours (Askari et al. 2018; Greenhow and Lewin 2016). For example, there is little evidence of the types of social media-based activities (e.g., selfies,^1^ hashtags,^2^ memes,^3^ likes,^4^ searches) that influence health behaviours (Dickinson et al. 2019). Most evidence has also been collected from surveys and/or questionnaires, and findings have according to Goodyear and Armour (2019) tended to be dominated by adult perspectives. Existing research has therefore tended to oversimplify the complexities of knowledge and behaviour change, using narrow measures (e.g. screen time) that often do not reflect the dynamic and interactive ways in which young people use social media.

## Theoretical framework and methodological considerations

In this paper we focus on learning in relation to social media. This focus allows us to purposefully step back from analysing the effects of social media use on health outcomes and to interrogate the multi-layered factors that shape how young people acquire new skills, knowledge and behaviours. While most studies seem to focus on the social media content, the effects of social media use, or the relation between content and effects, our interest is instead directed towards the mechanisms of the learning process, what sometimes is referred to as the ‘black box’ of learning (e.g. Epple, Argote, and Murphy 1996). We build on a methodological approach called Practical Epistemology Analysis (PEA) suggested by Wickman and Östman (2002). PEA is based in the works of Wittgenstein and Dewey, and it has been used and developed in numerous studies of learning and meaning-making as a way to explore meaning-making and learning processes as well as the direction that meaning-making processes (e.g. Lidar, Lundqvist, and Östman 2006; Lundqvist, Almqvist, and Östman 2009; Maivorsdotter, Quennerstedt, and Öhman 2015; Rudsberg and Öhman 2010). PEA has been used to analyse learning processes in-situ, but also on texts, drawings and interviews (Lundin and Jakobson 2014).

With inspiration from Dewey we in this paper focus on young peoples’ engagement with and experiences of social media. In a Deweyian transactional approach (Dewey and Bentley 1949/1991), knowing is not exclusively located in the minds of humans, but something practical, something that people do when acting in the world – and this focus on ‘doing’ and action is absent from much of the literature on young people, social media and health (see Dickinson et al. 2019). That is why Wickman and Östman (2002) use the term *practical* epistemologies (see also Quennerstedt 2013). Building on Wittgenstein, Wickman and Östman (2002) further argued that the meaning of something (e.g. a word, a thing) can be found in its use, and when a certain way to talk or act in relation to a certain question is privileged – that is a practical epistemology. The meaning of social media for young people accordingly lies in the transactional engagement and experiences of young people actual use of social media.

For Dewey, experience is: ‘ … the inclusive, multi-faceted, that is to say fully human, modes of prehending, reacting to, and interacting with our surroundings’ (Boisvert 1998, 14). This entails understanding experiences transactionally *in* the world rather than *of* or *about* the world. Experiencing is thus the ways a person act and engages with their surroundings (Dewey and Bentley 1949/1991).

It is not experience which is experienced, but nature – stones, plants, animals, diseases, health, temperature, electricity and so on. Things interacting in certain ways *are* experience; they are what is experienced. (Dewey 1929, 4a)

In this perspective experience refers to the different ways in which transactions take form in a specific situation and includes the experiencer as well as what is experienced (Jackson 1998). As Dewey argues: ‘An experience is always what it is because of a transaction taking place between an individual and what, at the time, constitutes his [sic] environment’ (Dewey 1938/1997, 43). To learn from experience is accordingly to connect the past, i.e. previous experiences, with the future, i.e. the direction of the experiences (Dewey 1916, 1938/1997). Every experience thus ‘influences in some degree the objective conditions under which further experiences are had’ (Dewey 1938/1997, 30). According to Dewey, we continually (trans)act and learn in experience, and learning is thus a functional coordination with the environment occurring all the time and sometimes in unexpected directions and ways (Garrisson 2001; Wickman and Östman 2002). Young people’s engagement with social media is in this perspective a continuous functional coordination between young people’s actions and experiences, social media content, the materiality of the device, other participants on social media etc., and according to Klaar and Öhman (2012) learning can then by using PEA be explored as a process ‘in which new relations to the environment contribute to extended possibilities for further action’ (441).

PEA was initially developed as a way to clarify mechanisms of learning, and to explore the progress of the learning process in relation to a certain purpose (Wickman 2012). By using the analytical concepts; purpose, gaps, relations, stand fast and encounters Wickman and Östman (2002) argue that learning in this way can be studied as ‘a process where gaps are filled by constructing difference and similarities in relation to what is immediately intelligible’ (603). PEA has often been used in studies closing in on educational practices in-situ looking at meaning making and what students are afforded to learn (Wickman 2012). We are interested in looking at a wider set of data and use the same concepts in order to identify specific experiences and interactions crucial for what a person does or does not learn, and thus how young people establish relations, distinguish evaluative entities and notice gaps in their engagement with health-related social media (see Lundegård and Wickman 2007). In this way we are not doing a detailed in-situ analysis of ongoing meaning making as in most PEA, but instead a more zoomed-out analysis where young people's experiences and engagement with health-related social media is in focus and where the analytical concepts of PEA are put to use. Questions that have guided us in this paper are:

- How is young people’s learning related to continuity and change in experience?
- How can the mechanism of learning be described?
- What influences the learning process?

In this way we can, as Wickman (2012) argues explore “the joint filling of *gaps* by the construal of *relations* to that which *stands fast* depending on *encounters* that occur … (146). The five key concepts of PEA that informed analysis in this paper are outlined in Table 2, and provided one way of investigating learning.

**Table 1.** Data collection methods.

Method	Description of the Method
Participatory Class Activities (n = 236, age = 13–15, m = 101, f = 135)	Young people worked in groups of 4–5 members, that were self-selected to support engagement in discussions. Each group completed a series of activities presented to them in an iBook. Data were acquired from 2 activities. ***Class Questionnaires (n = 236; m = 101, f = 135, age = 13–15):*** Each group watched a 2-minute video created by the researchers on current statistics of health-related social media use. Young people then individually completed a questionnaire, in the form of a leaflet. The class questionnaire was closed/open ended and was composed of 5 questions related to their uses of social media. Mean percentages were calculated for closed questions. Open-ended questions were categorised inductively and mean percentages were then calculated in relation to each category. ***Digital Pin Board (n = 53; m = 22, f = 29, mixed = 2 age = 13–15):*** A Pinterest digital pinboard was co-constructed with 10 young people during a pilot study on different types of health-related images and videos available on social media. From this process 55 images/videos were selected grouped into 11 categories: female body image, workouts, clean eating, sleep/mental wellbeing, motivation, physical activity, campaigns, male body image, governing body advice, commercial brands and celebrities. Inter- and intra- reliability tests were completed to confirm categorisation. A level of 85% was deemed appropriate (van der Mars 1989) and reached before the pinboards were used. In phase 2, each group was asked to either keep or delete the images. In turn, the pinboards provided data on the categories of health-related material young people attend to and would use. Mean percentage were calculated on the categories kept across all groups.
Focus Group Interviews (n = 84; age = 13–15, m = 35 f = 49)	19 focus group interviews (20–40mins) were conducted in the same groups from the class activities of 4–5 members. Two groups per class were interviewed (where possible). Groups were selected on the basis of offering a balanced sample on the health-related material young people access/attend to across the 10 schools and gender. Elicitation techniques were firstly used to encourage young people to discuss their pinboard. Semi-structured questions were then used to understand young people’s experiences of social media, and were common across all groups. The interviews complemented data obtained from the class activities

**Table 2.** PEA analytical concepts (from Lundqvist, Almqvist, and Östman 2009; Wickman and Östman 2002; Wickman 2012).

PEA Concept	Description of Concept
Purpose	Ascertaining the purpose(s) for the individuals’ participation or engagement in a particular activity and/or practice, asking what is going on here.
Gaps	Identification of gaps or indeterminacies in knowledge, behaviours, interactions and/or feelings. For example, a gap between what an individual desire and what they actually have in a certain situation.
Relations	Identification of what an individual does to ‘fill’ the gaps, and how they fill the gaps. For example, what information, practices, knowledge, skills etc., do individuals’ draw on to handle the indeterminacy. The overarching point is that individuals need to create relations between what they already know and the new situation, and that learning occurs when new relations are constructed.
Standfast	Identification of what is immediately ineligible and actions they use in a certain situation without hesitating what it means. A focus on what is not questioned and taken for granted when acting, and where no additional information is needed for the individual to address the gaps.
Encounters	Identification of what, who and in relation to what individuals meet and engage with in these situations.

## Research design

The data reported on in this paper is part of a wider project that investigated young people’s engagement with health-related social media, in the areas of physical activity, diet/nutrition and body image. An iterative mixed method research design was adopted to generate rich, in-depth and detailed insights into young people’s experiences of social media. Central to the research design was the active participation of young people, and a multi-method approach – involving class activities, interviews, a survey and workshops – was used to engage with multiple and varying sample sizes of young people at different stages.

### Ethics

A culturally responsive relational and reflexive approach to ethics was adopted (Sparkes and Smith 2014). This approach ensured that ethical decision-making was contextualised within the digital cultures and contexts inhabited by young people (Goodyear 2017). Following this approach, care for participants involved creating and using data collection methods that ensured participant safety, privacy and dignity, and that promoted participant autonomy. Furthermore, by adopting a responsive and reflexive approach, the research aimed to prioritise youth voice and expertise in data collection, where data collection methods were co-produced with youth (during pilot work) in an attempt to navigate research-youth power dynamics. University ethical approval was granted and informed consent or assent were obtained from participants. Legal conditions of social media were adhered to by using university accounts and devices, and ensuring participants were age 13 or over.

### Participants and setting

Overall, the fieldwork took place with 1346 young people (age 13–18) and was carried out in 10 UK schools, and across 12 classes (4 mixed gender, 3 boys only, and 5 girl only classes). Of the 10 schools, 2 were private, 3 were state and 5 were academies, 2 of which were faith schools.^5^ The schools were located in diverse socio-economic areas and included students from a range of different ethnic backgrounds. The findings are therefore representative of a diverse British population.

### Data collection

Data collection was framed by an initial focus on health-related social media in the areas of physical activity, diet/nutrition, body image and sleep. A conceptually narrow focus on health-related content was taken to provide clarity for participants and depth in the data on the types of health-related material young people engaged with, as well as the conditions of young people’s experiences. The data collection methods relevant to this paper are outlined in Table 1. This paper focuses on data collected from interviews. This decision was informed by the initial phase of data analysis of the wider project outcomes. It was apparent that the interviews provided in-depth insights into young people’s perspectives on how their engagement with social media shaped their health-related knowledge and behaviours. The remainder of this paper therefore focuses on the data generated from interviews. For information about the methods from the wider project see Goodyear and Armour (2019).

#### Interviews

19 focus group interviews (n = 84; age 13–15; m = 35; f = 49) were conducted and had the aim of providing a rich data set on the health-related information from social media young people engaged with, used, and/or disregarded. Two groups (4–5 members) per class were interviewed (where possible), and the groups selected were mixed gender (n = 7), boys only (n = 4) and girls only (n = 8). Prior to the interviews, the research design had involved participatory class activities (see Goodyear et al. 2019b) – see Table 1 – and young people were interviewed in the same groups. The data generated from class activities informed the sampling and content of interviews. In terms of sampling, a purposeful sampling strategy was adopted (Sparkes and Smith 2014); focus groups were selected on the basis of offering a balanced sample on the types of health-related material young people accessed/attend to across the 10 schools (identified from class activities, e.g. from unregulated/regulated material, commercial, peer/youth-based – see Table 1), as well as gender. In terms of the content of interviews, elicitation and semi-structured techniques were used. Elicitation techniques were firstly used to encourage young people to discuss the data generated from their group during the class activities. In particular, groups were asked to discuss the digital pinboard they had created during the Pinterest^6^ task. The aim of the Pinterest task was for groups to create a pinboard that they would associate with health. The Pinterest task had three activities: (i) groups selected health-related posts (‘pins’) to ‘keep’ or ‘delete’ from images/videos the research team had selected. The images/videos were common across all groups and contained eleven categories of images (see Table 1); (ii) to add any images/videos that the group felt were important, and that they would use; (iii) to name their Pinterest board. Following elicitation questions, semi-structured questions were used to provide further depth in the data on young people’s individual and collective experiences of health-related social media. The questions were common across groups and were focused on how young people engaged with health-related social media (what young people engaged with, used, and/or disregarded) and the perceived influence of social media on health-related knowledge and behaviours (in the areas of physical activity, diet/nutrition and body image). The interviews were led by a researcher and lasted between 20–40 minutes, as recommended for this age group, and to fit with the school’s timetable (see Burnette, Kwitowski, and Mazzeo 2017; Livingstone and Sefton-Green 2016). This interview duration is consistent with school-based focus group interviews conducted with young people in the area of health (Burnette, Kwitowski, and Mazzeo 2017; Harris et al. 2018).

### Data analysis

Data analysis was grounded in our guiding questions exploring how young people in focus groups describe their experiences of using health-related social media and thus how they make meaning of their engagement with social media. By using PEA to analyse young people’s descriptions of their experiences and engagement with health-related social media we can say something about how young people learn and what direction that learning takes. The analysis took place in two overarching phases.

In phase 1, all transcripts were read through by the first author to identify key segments of data that were representative of learning. In this phase two overarching descriptive segments were identified from the data: (i) Gymlads and (ii) Skinny girls. Gymlads is about young boys engagement with a social media-based gym culture involving images and videos of workouts. Skinny girls is about how young girls present their bodies on social media. Data that related to Gymlads and Skinny girls were then deliberated by the authors (see Goodyear, Kerner and Quennerstedt 2019c). Building on Tracy (2010) and Smith and McGannon (2018) guidance for conducting high quality research, the goal of this so called deliberative strategy (Goodyear et al 2019c) is a form of collective agreement, where all co-authors are given the possibility to make judgements in relation to different alternatives, views and arguments. This process identified distinct differences in relation to how boys and girls learn and in what direction their learning followed. As a result, and to generate further and in-depth understandings into mechanisms of learning, the data that related to either Gymlads and Skinny girls were grouped together to form two separate categories of data.

In phase 2, the two categories of data were analysed separately using PEA (see Andersson et al. 2018). Following on from established work that has used PEA, five concepts were used for in-depth analysis: (i) purpose; (ii) gaps; (iii) relations; (iv) stand fast and (v) encounters. These five steps are described in Table 2.

Five analytical questions derived from the PEA concepts were then deliberated, decided upon and used. The analytical questions related to each PEA concept were: (i) what is the purpose of young people’s engagement with health-related social media?; (ii) what gaps are identified in their experiences of social media?; (iii) what do young people do, use, or draw upon to ‘fill’ the gaps?; (iv) what is obvious or taken-for-granted for young people in relation to health-related social media?; and (v) what issues or questions do they engage with in relation to the identified gaps? The grouped data sets for Gymlads and Skinny girls were independently coded and categorised by both authors using the five analytical questions. The deliberative strategy (Goodyear et al 2019c) was then applied, and the authors discussed their analysis in relation to each question, with the aim of making something (i.e. a coherent story) ‘in common’. Once the co-authors had reached a collective agreement, the deliberative strategy was extended beyond the authorship team to further develop a level of analytical rigour. Eight sport pedagogy researchers with different subject expertise (e.g. policy, history, social justice, assessment) and career experience (e.g. PhD researcher, post-doc, early career, senior and professor) were provided with a representative interview transcript. The eight researchers read the transcript independently and then met as a collective group with the co-authors to discuss the data in relation to evidence of learning. This process provided different alternatives, views and arguments, and enabled the authors to further establish ‘common’ interpretations of the data set with individuals who were not actively involved in the research project.

### An empirical short story

In the results section, both a more zoomed-out perspective and a zoomed-in empirical short story of the data is provided in relation to the five PEA concepts. The PEA analysis, and findings from the wider project in which this paper is situated (see Goodyear and Armour 2019) provide evidence on the complexity of young people’s uses of social media, and the very clear gaps that exist between adults and young people’s understanding of social media. In this context, stories are particularly useful as they have the advantage of providing thick descriptions and facilitating rich interpretations of key findings (Phoenix, Smith, and Sparkes 2010; Smith et al. 2013). Short stories are furthermore relevant to this paper as this genre of storytelling is often used to present complex issues in the format of a self-contained incident or series of related events (Gilbourne, Jones, and Jordan 2014). Accordingly, and grounded in concepts of naturalistic generalisability and transferability (see Smith 2017), an empirical short story was selected as a key strategy to help readers understand the complexity of young people, social media and learning (naturalistic generalisability), and to help readers engage with the findings in relation to their own contexts (transferability).

The short story reported in this paper is derived from the data that were placed into the category of Gymlad. The short story is of one of the 19 interview transcripts, and is of 5 year 9 boys (age 13–14). By focusing on one transcript and one group of boys when presenting the results of the more zoomed-in perspective, in-depth insights are provided into the five PEA concepts and, in turn, mechanisms of learning. The selected short story was deemed by the co-authors to be representative of the wider data set, so in this sense it both represents the larger set of data and provide the possibility to present the results in detail.

## Results

### Learning – a zoomed out perspective

Before illustrating the short story we demonstrate how the short story is representative of the wider data set on how boys use health-related social media i.e. a zoomed out perspective. Data reported across all of the interview transcripts – that included both boys and girls – provide evidence that social media is always present for most boys in the study – everyone is on social media. The health-related social media they engage with involves health being understood as exercise like going for runs, rock climbing or football, but mostly about being fit in terms of diet and working out in the gym. In their engagement health and fitness becomes a matter of looking alright, striving for being ‘ripped’ with ‘six packs’ and getting bigger – #gains – but at the same time being ‘slim’. Being overweight is positioned as not looking good and thus being unhealthy. So healthy eating becomes important and sport is sometimes described as good because you burn calories by doing sport.

Engaging with pictures and videos of workouts on social media motivates them to exercise. The visual media is sometimes of really fit people making them jealous, wanting to be like that person, but also if it is normal people doing normal things and not being too extreme. Celebrities are also important but not for them, just for others. At the same time they experience a constant pressure to look a certain way, to do different things, and to be a certain way, seeking confirmation from other people on social media in order to see what they think of them. They also experience being at risk when they post on social media with fears of shaming, getting bantered^7^ or even bullied. When presenting oneself as a healthy person in a confessional space like social media fitness becomes a way of fitting in. However, even if shaming is a constant risk, humour and irony is an essential aspect of their engagement with health related social media. For example #Gymlad becomes an ironic hashtag for a person, group but mostly of other people, of ‘gym guys’ showing off their six packs.

### Learning – a zoomed in perspective

In this section we report on the data from the empirical short story that resulted from the PEA analysis using two segments of the data. The short story as a whole is representative of two different purposes of adolescent boys uses of health-related social media in the whole data set: (i) communication with friends, and (ii) accessing health-related information. Data are reported verbatim, with only short responses (represented by …) such as yes, yep, agree omitted. A descriptive analysis is also provided using PEA concepts, where we refer to specific quote line numbers (e.g. Q1)

#### Communication with friends

1. It’s just the thing – in our generation, everybody has got Instagram and Snapchat and it’s just easy to find, and use it, and communicate with everyone.
2. I use WhatsApp as well but that’s mainly for homework and family chats, that’s really it.
3. And how often do you use social media?
4. Basically, every day … .
5. A lot, probably every hour I’ll check Snapchat.
6. Every time I get a notification really.
7. So, it gives you a notification and then you’ll go on and check it?
8. Yes.
9. I’ll check it even if I don’t have a notification; I’m just hoping it’s glitched – I have a message. (Laughs).
10. Please let me have a message. I want to feel popular!
11. Do you guys post about health and wellbeing?
12. I do, on Instagram and Snapchat.
13. I’ve sent pictures to my close friends.
14. Of your six pack? (Laughs).
15. No, just stuff I’m doing at the gym but not publicly.
16. I’ll do that sometimes too. I’ll be on the way to rugby training or something when I’m [snapping 0:01:48] someone … .
17. I don’t put it on Instagram. I don’t think I have the confidence to do that really. I feel like I’d get bullied, I’m not going to lie.
18. Yes, you would be, but it would be jokingly, I reckon.
19. No, it probably wouldn’t.
20. Why would you get bullied for posting a picture of food?
21. No, I mean if I posted [** 0:02:28]
22. Oh, gym lad, #gains.
23. Why is it you don’t think the reaction of other people would be very positive?
24. It would be joking around. For a week or so you’d get roasted, and then it would just go dead … .
25. If someone does something funny or out of place, that’s the joke at the moment. It will go around for a few weeks maybe and then people forget about it. It’s just a funny in-joke.
26. Do people comment on the stuff that you post, like your diet stuff? …
27. One girl I know, she’s ripped, she posts stuff from the gym because she had an operation. She’s 10 months post-op. And then diet, she’ll post her ‘avocado, egg on toast’. She actually posted that, that’s her, she’s pretty atypical really.
28. I’ve seen some guys our age posting topless pictures if they’re ‘ripped’ or whatever, but not everyone, it depends on their confidence.
29. What do you do if you see those kinds of pictures?
30. Stare in admiration.
31. Like’ it and be jealous. (Laughs) … .
32. You guys also mentioned something about gym lad about two minutes ago. What does gym lad mean?
33. About two years ago everyone was using the #bitofagymlad. It was like an ironic thing. It was anytime anybody did anything remotely athletic, they posted #bitofagymlad.
34. What would you a say a gym lad is?
35. A guy who goes to the gym.
36. A lad who goes to the gym.
37. Pretty much. You can either use it ironically or unironically, I guess. It’s mainly ironic.
38. Yes, you’d get roasted if you put unironically #Imagymlad.

The boys use social media for the *purpose* of communication, with friends and family members, and to exchange posts about homework and health and wellbeing. This *purpose* opened up *gaps* between the body the boys currently have and the body they desire, where they become jealous (Q31) because they see posts by people they know (Q27) and/or of a similar age (Q28). The boys fill these gaps with *relations* by either avoiding posting about health and wellbeing in public spaces (Q13, Q15), such as Instagram (Q17) or by using irony when they post images about their bodies (Q37), using the hashtags #gains (Q22), #bitofagymlad (Q33) or #Iamagymlad (Q38). These relations are significant because related to the purpose of communication, feeling popular on social media is important to the boys (Q10) where they are concerned for other people’s feedback, and were fearful of being bullied (Q17) and/or getting roasted (Q24). In these situations, the boys *encounter* is with a discourse of health and fitness, that takes place in public and private spaces of social media, where everybody should do better and strive for a more fit, slim and muscular body. What *stands fast* is the notion that a ripped body and looking good equates to health (Q27, Q28), and that health is something that should and could be presented on social media. Also the very notion of being ‘on’ social media is not questioned (Q1).

#### Accessing health-related information

(39) When I first got in to exercise, it was because I saw a post on Instagram – a simple workout you can do every morning. So I started doing that and I got more in to it.
(40) I started because there was this guy at my gym and I thought ‘Damn he’s ripped,’ and then I thought ‘Hey, I’m not.’
(41) And you’re still not!
(42) Pretty much! He’s like the goal, pretty much.
(43) Yes, goals is the thing.
(44) That was why I started, because of him.
(45) Do you think the posts on social media to do with health and wellbeing can be helpful for your health?
(46) Yes.
(47) Yes.
(48) Also, bad, as well, really bad … .
(49) The Beckham one wasn’t impressive.
(50) They were veins.
(51) They’re so vascular, I don’t even like it.
(52) I think a little bit of vascular is fine.
(53) There are different preferences here; I’m not a big fan of the vascular.
(54) If the veins pop out, it puts me off.
(55) It’s weird. I talked to my mum about this the other day because we went to the gym together, and she saw this one guy and he was ridiculous, and my mum said ‘Ugh no!’
(56) The one with Brooklyn Beckham, I remember that one, he wasn’t even that ripped.
(57) And his hair wasn’t on point. (Laughs).
(58) Yes, it wasn’t anything; it wasn’t his style.
(59) Brooklyn Beckham is a bit of a lad, but that picture wasn’t his best.
(60) Okay, so that kind of links to celebrities as well; do you guys think that celebrities have an influence to do with health and wellbeing?
(61) Yes, I follow three celebrities, like D Double E (a rapper), Anthony Joshua and a few others who posts videos of themselves working out, so, I get inspired off that as well.
(62) I follow a lot of weightlifters and stuff because that’s quite inspiring
(63) I follow quite a few sportsmen. I’m a bit disappointed because I was expecting some sort of advice, but it’s just videos of them doing stuff.
(64) You have the rapper, but do they tend to be sportspeople generally? Is that fair, or are there a lot of celebrities who are reality stars? …
(65) Yes, on YouTube, some people just post videos of them going to the gym; that’s kind of good as well … .
(66) I used to find articles online from Men’s Health magazine, or whatever, until I realised that generally, there’re not [** 0:13:09]
(67) They’re not even that great.
(68) And also, it’s just a picture of a massive guy with ridiculous veins and muscles. I’d just rather make my own workout.
(69) And the ones that you watch, do you tend to watch with a specific person? Because there are YouTubers aren’t there?
(70) Yes.
(71) Yes, it’s not exactly a specific person. The ‘suggested for you’, if I think it’s kind of cool, then I just watch it.
(72) I go on the ‘trending’ and I find pictures. Sometimes I’ll find something by a specific YouTuber, and then I’ll get recommended a bunch of their other videos and I’ll go through a few days just repeatedly watching their videos and then get bored and find someone else.
(73) You can get an infinite loop of YouTube. I might just start watching one video and then another interesting video will pop up and you’ll watch that … .

The boys used social media for the *purpose* of accessing information on exercises and/or gym workouts to help them in becoming ‘ripped’ (Q39, Q40). This purpose opened up a *gap* in relation to a lack of advice on from some of the social media videos. In particular, videos from sportsmen (Q63), and accounts associated with magazines (Q66, Q67, Q68) were not considered to be informative. Some of the celebrity accounts – such as Brooklyn Beckham – were also considered to provide information that was irrelevant to their bodies, in terms of the posts being too ‘vascular’ (Q51) where the ‘veins pop out’ (Q54), and this was despite their credibility as being ‘a bit of a lad’. The boys filled these gaps by accessing videos from YouTube of people exercising in the gym, who were not specific people (Q71) such as celebrities, sportsmen or reputable exercise and health organisations. The videos were those that were trending (Q72) and that were ‘suggested’ (Q71) and could be played on ‘loop’ (Q73). Similar to the purpose of communication with friends, in these situations the boys *encounter* is again with a discourse of health and fitness where health can be achieved through being fit in terms of diet and working out in the gym. What *stands fast* is the notion that a ripped body equates to health, which was seen as a reasonable as well as valuable goal to achieve (Q27, Q28).

## Discussion

This paper reports new evidence on how young people learn in relation to social media. New evidence is provided on the mechanisms involved in adolescent boys learning about their bodies, exercises and workouts, and the mediating and moderating factors of social media use that shape the direction of learning. Two main purposes of adolescent boys engagement with health-related social media were identified; (i) communicating with friends, and (ii) accessing health-related information. Previous studies have shown that young girls and boys use social media in different ways and for different purposes (Booker, Kelly, and Sacker 2018; Ofcom 2017), where there is evidence that girls are primarily interested in the social networking aspects of digital media, whereas boys tend to be more interested in interactions associated with their hobbies and interests (Booker, Kelly, and Sacker 2018; Ofcom 2017). The purposes of the boys engagement on social media in this study are related to both interest and the social networking aspects of social media.

The data revealed that irony and humour were the central learning mechanisms for the boys in the study. It has been reported previously that irony is typically represented in social media through hashtags, memes and GIFs (DeCook 2018; Page 2019). In this paper, irony and humour were present within the boys uses of hashtags, such as, #bitofagymlad #gains #Iamagymlad, as well as how the boys made judgements about credible information, such as in the accounts of Brooklyn Beckham as a ‘bit of a lad’. These hashtags and meanings represented a certain kind of genre of communication amongst the boys, that those external to this media culture may not understand. Indeed, irony is complex to navigate as it is the basis for mismatch, whereby a positive comment also expresses negative criticism (Andersson 2010; Page 2019). In this paper, the boys’ uses of irony made conflicting perspectives about the body visible, and provided a basis for the boys to understand the situation, evaluate their bodies and know how to proceed within a complex social hierarchy of ‘ideal’ bodies as present within their peer networks on social media.

It was apparent in the data that the boys uses of irony and humour were connected to self-presentation and positive impression management. Feelings of popularity on social media were of central importance to the boys and the use of positive irony (see Page 2019) was a key strategy that enabled the boys to participate within the online gym culture. The data showed that the boys posted to social media about their athletic activities, where the accompanying hashtag – #bitofagymlad – cancelled out the potential for unfavourable evaluations of the body. There is evidence from previous research on #uglyselfies that positive irony is an effective positive impression management approach (Page 2019). Ironic hashtags tend to navigate ridicule and result in more praise and comments from others (Page 2019). Furthermore, the more humorous a post – such as #Iamagymlad – increases likeability (Page 2019). It can therefore be argued that feedback and praise are key sub-learning mechanisms that were present in this paper and that relate to irony and humour.

The boys skilful uses of social media were also evident from the different ways in which they used the public and private spaces to post about their bodies. Ironic hashtags were used to post in public spaces, whereas literal posts about their bodies were only shared within private spaces of social media. Extending previous understandings of irony and the affective influence of hashtags, memes and GIFs on social media users (see Andersson 2010; DeCook 2018), the data indicate that the hashtags were an affective form of content, that, in-line with the action-orientated approach of PEA, we refer to as an affective action. In this way, the boys used irony and humour to participate within health-related social media, and in a way that enabled them to engage with, uphold, and handle health discourses associated with masculinity – such as being ripped – without fear of ‘literal’ peer ridicule and within a context of acceptable ‘banter’.

Beyond irony, humour, feedback and praise, the data revealed the embodied and interactive aspects of learning processes, as well as the mutuality of structure and agency within social media(Goodyear et al 2019b; Andersson, 2010).There was evidence that young people’s engagement with social media was a continuous functional coordination between young people’s actions and experiences, social media content, and the materiality of the device and other participants in social media. In particular, the adolescent boys developed expertise via their participation in peer-based social media contexts, and through the use of multi-modal resources, in which learning was self-initiated as well as peer-influenced. For example, by generating and interacting with content related to workouts and the body, with other participants and via images and hashtags, young people learnt about their own bodies, how to access usable health-related information, and how to navigate against negative feelings of self.

The data generated provide additional evidence for the importance of investigating learning processes, in order to understand the direction and form of learning. For example, knowledge related to ideas about healthism, in terms of health equating to fit, slim and/or muscular bodies were proceeded without any hesitation. It was in many senses taken for granted. This finding suggests that the boys had already learnt of discourses associated with healthism, thus presenting challenges to much of the evidence reported on the relationship between social media content and effects, that is particularly prevalent in research focused on social media content and body image, such as #fitspiration (Burnette, Kwitowski, and Mazzeo 2017; Rich 2019). Drawing on work within critical health education and pedagogy, it can be suggested that the encounters examined in this paper illustrate social media as a health-related instructional pedagogy (Rich 2019), where messages about how to regulate the body are deeply infused with a ‘corporeal ethic, a socially regulative moral code’ (Evans and Rich 2011, 365). Hence, the data reported in this paper present new challenges to the content-effects literature by highlighting the complexities of the ‘black box’ of learning in relation to different directions in learning.

The PEA approach adopted and illustrated in this paper open ups new ways of investigating learning within the context of social media. To make claims about learning, the PEA approach we adopted was explicitly informed by learning theory – namely Dewey – and we hope that this theoretical alignment with methods (see for further detail Quennerstedt, Öhman, and Armour 2014) will inspire future studies to make stronger use of learning theory when investigating learning in relation to social media. The methods used to generate data for PEA analysis could also be further developed. In this paper we are well aware that the focus group interview situation is a different transaction compared to actual social media use, and in a sense second hand information about boys’ actual engagement with social media. However, by focusing on adolescent boys’ experiences of social media and the meanings they make in communication about their social media use we would argue that we can make an important contribution to the question of how young people learn in relation to health-related social media, and what direction this learning takes, i.e. how ways of learning influence the route learning takes. That said, there is space for the application of in-situ methods, such as examining ‘moving methods’ using GoPro’s to interview in-action (Palmer 2016) and this would facilitate the development of more action-orientated knowledge – *practical* epistemologies – in relation to learning.

## Conclusion

To date, there has been too little attention into how young people learn in relation to social media (Askari et al. 2018; Greenhow and Lewin 2016). Most evidence reports on risks oversimplifying the complexities of the learning *process* because research has tended to be grounded in the effects of use (e.g. physical inactivity, wellbeing – Stiglic and Viner 2019), the relationship between effects, time and content (e.g. screen time – Orben and Przybylski 2019), and/or focused on contextual factors influencing effects, time and content (e.g. assemblages and/or relationality with friends, home, school and social media – see Rich 2019). In this paper, we provide evidence that PEA generates new ways of understanding aspects of learning, by focusing on mechanisms of learning in-action, and in relation to what young people do on social media – a zoomed-in perspective. Hence, by looking at ‘the joint filling of gaps through relations’ (Wickman 2012, 146), PEA is an approach that enables researchers to answer particular questions about how young people acquire knowledge, and make practical, moral or aesthetical meaning in relation to their engagement with social media. The findings from this paper make an original contribution to knowledge by providing new evidence on young people’s social media-based actions relative to learning – such as irony and humour. There was evidence that young people were critical users and generators of social media information and mediums, who are clearly thinking through what they see, do, and use online. At a time when the narratives associated with young people and social media are dominated by risk, this paper provides a fresh evidence-based perspective also on the potentially positive role of social media as a health-related learning resource in young people’s lives. This evidence can be used to inform evaluations of social media use, the design of educative support for young people, and guidance for teachers, parents and professionals/practitioners to help these adults better understand young people’s uses of social media and the support they require.

## Notes

1. A selfie is a self-portrait type image, typically taken with a smartphone which may be held in the hand or supported by a selfie stick.

2. A hashtag is a type of metadata tag used on social networks such as Twitter and other microblogging services, allowing users to apply dynamic, user-generated tagging which makes it possible for others to easily find messages with a specific theme or content.

3. An image, video, piece of text, etc., typically humorous in nature, that is copied and spread rapidly by Internet users, often with slight variations.

4. A like button, like option, or recommend button is a feature in communication software such as social networking services, Internet forums, news websites and blogs where the user can express that they like, enjoy or support certain content.

5. For an overview of the characteristics of different types of schools in the UK please see https://www.gov.uk/typesof- school.

6. Pinterest is a social media booking marking site that enables the discovery and sharing of information – please see https://www.pinterest.co.uk .

7. The playful exchange of teasing remarks .
